# Spatial Omics in Clinical Research: A Comprehensive Review of Technologies and Guidelines for Applications

**DOI:** 10.3390/ijms26093949

**Published:** 2025-04-22

**Authors:** Yoonji Lee, Mingyu Lee, Yoojin Shin, Kyuri Kim, Taejung Kim

**Affiliations:** 1 College of Medicine, The Catholic University of Korea, 222 Banpo-daero, Seocho-gu, Seoul 06591, Republic of Korea; gina228@catholic.ac.kr (Y.L.); mkl0107@catholic.ac.kr (M.L.); yoojins07@catholic.ac.kr (Y.S.); 2 College of Medicine, Ewha Womans University, 25 Magokdong-ro 2-gil, Gangseo-gu, Seoul 07804, Republic of Korea; kkyuri01@ewhain.net; 3 Department of Hospital Pathology, Yeouido St. Mary’s Hospital, College of Medicine, The Catholic University of Korea, 10, 63-ro, Yeongdeungpo-gu, Seoul 07345, Republic of Korea

**Keywords:** spatial omics, spatial transcriptomics, spatial proteomics, spatial epigenomics, spatial omics data analysis tools

## Abstract

Spatial omics integrates molecular profiling with spatial tissue context, enabling high-resolution analysis of gene expression, protein interactions, and epigenetic modifications. This approach provides critical insights into disease mechanisms and therapeutic responses, with applications in cancer, neurology, and immunology. Spatial omics technologies, including spatial transcriptomics, proteomics, and epigenomics, facilitate the study of cellular heterogeneity, tissue organization, and cell–cell interactions within their native environments. Despite challenges in data complexity and integration, advancements in multi-omics pipelines and computational tools are enhancing data accuracy and biological interpretation. This review provides a comprehensive overview of key spatial omics technologies, their analytical methods, validation strategies, and clinical applications. By integrating spatially resolved molecular data with traditional omics, spatial omics is transforming precision medicine, biomarker discovery, and personalized therapy. Future research should focus on improving standardization, reproducibility, and multimodal data integration to fully realize the potential of spatial omics in clinical and translational research.

## 1. Introduction

Cells, the fundamental units of life, are essential in understanding the structure, function, and behavior of living organisms [1]. To gain deeper insights into cellular biology, various technologies have been developed to detect and study cells over the past decades. Conventional single-cell technologies, such as single-cell RNA sequencing (scRNA-seq), have enabled the identification of cell types by profiling the entire transcriptome from thousands of individual cells [2]. These advances have also facilitated the creation of cellular reference maps for various organisms that capture the position, function, and characteristics of each cell type [3]. However, despite these breakthroughs, conventional techniques often fail to retain critical spatial information, leading to the loss of essential context regarding cellular organization and interactions. To overcome this limitation, spatial omics technologies have emerged, allowing the measurement of molecular characteristics of cells while preserving their native spatial context [4]. These innovative technologies allow the precise localization of cells within tissues while simultaneously providing quantitative measurements of gene expression directly in situ [5,6].

This review provides a comprehensive exploration of the diverse landscape of spatial omics, encompassing transcriptomics, proteomics, and epigenomics. For each domain, commonly used technologies will be introduced, followed by a detailed discussion of their respective strengths and limitations. In addition, methods for analyzing the complex and large-scale datasets generated by these techniques will be highlighted. Finally, the applications of spatial omics will be examined in various clinical contexts, with a focus on cancer research, neurological diseases, and autoimmune disorders. By offering an in-depth overview of how these technologies are advancing biomedical research and transforming our understanding of health and disease, this review aims to emphasize the potentially critical role of spatial omics in modern life science.

## 2. Spatial Omics and Technologies

### 2.1. Spatial Transcriptomics

Spatial transcriptomics is a powerful method that enables researchers to spatially localize and quantify gene expression in the form of mRNA transcripts within cells or tissues that are in their native state. Traditional RNA sequencing methods, such as bulk RNA sequencing, which averages gene expression across all cells in a tissue sample, obscures cellular heterogeneity and lacks spatial context. ScRNA-seq involves dissociating cells from tissue, allowing for high-resolution analysis of individual cell types, but at the cost of losing positional information, cell interactions, and cell organization across the tissue landscape [7,8]. In contrast, spatial transcriptomics preserves spatial information by analyzing intact tissue sections, maintaining insights into both gene expression and tissue architecture [9]. Furthermore, it can elucidate molecular interactions between tissue components, uncovering mechanisms behind non-autonomous cell phenomena, such as tumor interactions with the surrounding microenvironment, immune cell infiltration within tissues, and the establishment of developmental gradients [10].

Spatial transcriptomics technologies can be grouped into two main categories: next-generation sequencing (NGS)-based approaches and imaging-based approaches. NGS-based methods capture RNA locally from intact tissue sections on a pixelated, DNA-barcoded surface and read out their gene identities using next-generation sequencing. Imaging-based methods rely on fluorescence in situ hybridization (FISH) or direct in situ sequencing, using multiple rounds of imaging and combinatorial barcoding strategies to visualize and quantify transcripts at single-molecule resolution [10].

#### 2.1.1. Next-Generation Sequencing-Based Methods

NGS-based methods enable the direct capture of RNA molecules from tissue samples. These approaches utilize a spatially barcoded surface, such as a slide or chip, either to capture polyadenylated RNA molecules directly from tissue samples or to transfer barcoded oligonucleotides onto the tissue.

The primary advantages of NGS-based methods are their scalability and the ability to capture mRNA in an untargeted manner. By adjusting the size of the capturing surface, these methods can accommodate a wide range of tissue sizes without significantly impacting data acquisition timelines. In addition, the untargeted capture of RNA from tissue slices eliminates the bias associated with predefining gene targets. Meanwhile, the relatively lower spatial resolution compared with imaging-based methods is partially attributed to lateral diffusion during mRNA transfer, which poses challenges in achieving single-cell resolution [11].

To further illustrate the applications and capabilities of NGS-based methods, the following section will delve into specific technologies, including Visium, Slide-seq, Stereo-seq, and DBiT-seq, highlighting their unique features, advantages, and limitations. Additionally, Table 1 provides a side-by-side comparison of these technologies for a summary and an at-a-glance assessment.

Visium (10× Genomics): the Visium platform is designed for whole transcriptome spatial profiling, providing a broad coverage of gene expression analysis across tissue sections [12]. This technique is compatible with various tissue types, including formalin-fixed paraffin-embedded (FFPE) and fresh frozen (FF) tissues. A key feature that distinguishes Visium from other platforms, such as Slide-seq, is its use of spatially barcoded reverse transcription (RT) primers printed directly onto glass slides. The workflow begins with tissue sections placed on a slide covered with more than 5000 spatially barcoded spots, each precisely positioned by a microarray spotting robot [10] and containing RT primers. Each barcoded spot has a 55 µm diameter, optimizing the resolution to distinguish histological structures and cellular features with high precision [13]. The capture area on the slide can range in size, available as 6.5 mm × 6.5 mm or 11 mm × 11 mm, accommodating different tissue sizes and research needs. Once the mRNA in the tissue sample binds to the barcoded primers, cDNA synthesis occurs. The spatially barcoded cDNA is then processed through sequencing library preparation, allowing for the simultaneous capture of up to 20,000 gene expression across an entire tissue section.

Visium also offers flexible assay options tailored to specific research needs, including HD Spatial Gene Expression, Spatial Gene Expression v2 and v1, and Spatial Gene+Protein Expression assays. The HD format offers a resolution of $2\;\mu m^{2}$, which is particularly valuable for studying detailed cellular architecture, while the v2 system—optimized for clinical translational research using archived specimens—offers 4× higher throughput. The Gene+Protein variant provides critical insight into gene–protein regulatory networks within preserved tissue contexts. Since the Visium platform can map gene expression across tissues with broad spatial resolution, it is well-suited for research that requires understanding large-scale spatial patterns, such as oncology [14] and neuroscience [15].

Slide-seq: Slide-seq is a technique in which RNA from tissue sections is transferred onto a surface covered with DNA-barcoded beads instead of barcoded RT primers. Each bead has a known spatial location, allowing researchers to determine RNA positions through sequencing. This method enables scalable, spatially resolved gene expression profiling at the whole transcriptome level [16]. The positions of the beads are not predetermined as they are randomly distributed. Consequently, their barcodes must be decoded in situ prior to sample preparation. This is achieved using Sequencing by Ligation (SBL), where probes bind to the barcoded beads, undergo ligation, and are subsequently detected via fluorescence. This process identifies both the positions of the beads and their associated barcode information [17].

The beads that are 10 µm in diameter serve as a solid support for the synthesis of oligonucleotides, with bead barcodes created by split-pool cycles, a process first developed for single-cell barcoding [10]. Slide-seqV2 is the latest advancement in Slide-seq technology, designed to enhance the original method. This version combines improvements in library generation, bead synthesis, and array indexing to reach an RNA capture efficiency of about 50% that of scRNA-seq data (10-fold greater than Slide-seq), approaching the detection efficiency of droplet-based scRNA-seq techniques [18].

Stereo-seq (SpaTial Enhanced REsolution Omics-sequencing): Stereo-seq utilizes DNA nanoball (DNB) technology for in situ capture of gene expression. DNBs are circular DNA molecules generated by rolling circle amplification (RCA), an isothermal process that generates long single-stranded DNA from a circular template [19]. Each molecule contains a Coordinate Identity (CID) sequence that encodes its spatial position. After generation, DNBs are fixed onto grid-patterned array chips, achieving a high-density arrangement. Each DNB measures 220 nm in diameter, with a center-to-center distance of 500 nm, enabling highly precise subcellular resolution [20]. However, Stereo-seq is currently limited to fresh frozen tissue, which restricts its use in certain clinical and archival studies. Despite this limitation, Stereo-seq enables whole transcriptome profiling and has been widely applied in various fields, including organ research, evolutionary biology, and disease modeling, but is particularly well-suited for developmental biology [21]. By mapping complex cellular interactions during developmental stages at the molecular level, Stereo-seq is poised to significantly advance our understanding of developmental processes.

DBiT-seq (Deterministic Barcoding in Tissue sequencing): DBiT-seq enables simultaneous mapping of mRNAs and proteins through microfluidic barcoding. This technology is compatible with FFPE or FF sections at near-single-cell resolution. DBiT-seq utilizes parallel microfluidic channels to deliver DNA barcodes to the surface of a tissue slide in a grid-like pattern. Two sets of barcodes (A1–A50 and B1–B50) are delivered, creating unique barcode combinations for each tissue pixel. This approach enables the generation of spatially resolved transcriptomic and proteomic profiles at adjustable pixel sizes of 10 μm, 25 μm, or 50 μm, depending on the desired resolution. Following barcode delivery, tissue is lysed, and spatially barcoded cDNAs and antibody-derived tags (ADTs) are collected for NGS. The spatial coordinates of gene and protein expression are reconstructed based on barcode combinations, allowing for high-resolution mapping across the tissue [22]. By enabling simultaneous multiplexed transcriptomic and proteomic profiling, DBiT-seq offers a powerful multi-omics platform to study cellular heterogeneity and tissue organization [23].

#### 2.1.2. Imaging-Based Methods

Imaging-based spatial transcriptomics methods utilize fluorescence microscopy combined with specific probes that hybridize to target RNA molecules, enabling direct visualization of gene expression without requiring RNA extraction. Imaging-based methods are inherently targeted, relying on predefined gene sets for analysis [10], while ISS-based approaches and whole transcriptome methods offer untargeted profiling, capturing RNA molecules without prior selection of targets. Recent advances in multiplexing and detection efficiency have significantly increased their utility and throughput, allowing the detection of more than 20,000 genes [6,24].

Historically, fluorescence-based RNA detection in situ has been constrained by spectral limitations, which restrict simultaneous imaging of more than 5–10 fluorescent molecules. Recent breakthroughs in multiplexing have overcome these challenges through sequential rounds of hybridization and imaging, enabling the simultaneous detection of multiple RNA species. Consequently, imaging-based methods are now largely defined by their detection modalities, the labeling strategies for RNA molecules, and the multiplexing techniques employed to detect multiple RNA transcripts across sequential imaging rounds [10].

Imaging-based methods can be broadly categorized into two main subtypes: in situ hybridization (ISH) and in situ sequencing (ISS) methods [17,25]. ISH strategies hybridize labeled probes directly with target RNA molecules within intact tissue, allowing visualization of transcripts in their original tissue context. This approach provides high sensitivity and spatial resolution, which makes it ideal for analyzing specific RNA molecules and their subcellular localization [6]. ISS methods perform RNA sequencing directly on tissue sections, typically by ligation (SBL), gene barcodes (targeted), or short fragments of cDNAs (untargeted) in situ [26]. By combining RNA detection with sequencing, ISS allows spatially resolved transcriptomic analysis at the single-cell level. Compared with ISH techniques, this approach also has the ability to detect single nucleotide variants (SNVs) [17]. These methods are summarized by detection principle, resolution, multiplexing capacity, tissue compatibility, and key applications in Table 2.

Xenium (10× Genomics): the Xenium platform is a cutting-edge in situ transcriptomics technology provided by 10× Genomics, capable of detecting and mapping gene expression patterns at subcellular spatial resolution. It is designed to analyze hundreds to thousands of RNA targets within FFPE and FF tissues [27,28]. By providing high-resolution data, Xenium is particularly suited for studying tissues with spatially heterogeneous gene expression, such as the brain [29] or tumor microenvironments (TMEs) [30].

The workflow begins with a 10 µm tissue section placed onto a Xenium slide. Xenium employs padlock probes, circularizable oligonucleotides that hybridize to specific RNA targets. Upon ligation, these probes enable RCA to enhance signal detection. The slide is then processed in the Xenium Analyzer, which performs multiple cycles of fluorescent probe hybridization and high-resolution imaging. Each gene is assigned a unique optical signature, enabling precise decoding of target genes and the generation of a spatial transcriptomic map across the entire tissue section [31].

Xenium employs FISH to achieve exceptional sensitivity and specificity in detecting individual RNA molecules. This high spatial resolution enables detailed mapping of gene expression at the subcellular level, offering critical insights into cellular organization and function within tissue contexts. The multiplexing capability of the platform further allows simultaneous quantification of up to 5000 genes within the same cell, facilitating comprehensive analyses of cellular heterogeneity and interactions [30,32].

MERFISH (Multiplexed Error-Robust Fluorescence In Situ Hybridization): MERFISH is an RNA imaging technology that extends the capabilities of single-molecule FISH (smFISH) to enable spatially resolved, massively multiplexed profiling of gene expression at a single-cell resolution of 0.1 µm. While smFISH employs fluorescently tagged oligonucleotide probes to label RNA transcripts of interest and determine gene expression by directly counting fluorescent signals, MERFISH significantly enhances this approach by integrating combinatorial labeling, sequential imaging, and error-robust barcoding to analyze thousands of RNA species within individual cells [20,33].

The MERFISH workflow involves labeling each gene with a unique binary barcode consisting of a series of “0”s and “1”s. Each barcode is designed to contain a predefined number of “1”s (typically four), strategically distributed in multiple rounds of imaging. This design minimizes optical crowding and allows for precise error detection and correction [20]. Unique oligonucleotide probes are designed for each RNA target, containing a target-binding region complementary to the RNA sequence and hangout tails that hybridize with secondary readout probes. Fluorescently labeled readout probes are introduced in successive imaging rounds, where the presence of fluorescence is recorded as “1” and its absence as “0” [11,20].

Throughout this workflow, MERFISH preserves spatial context, avoids amplification biases, and enables high-resolution transcriptome analysis while maintaining high multiplexing capacity [33]. Its applications span diverse fields, including developmental biology [34,35], neuroscience [36,37,38], and cancer research [39].

SeqFISH+ (Sequential Fluorescence In Situ Hybridization): seqFISH+ achieves super-resolution imaging and enables multiplexing of over 10,000 genes in single cells. By employing sequential hybridization and imaging with a standard confocal microscope [40], seqFISH+ facilitates high-throughput, spatially resolved transcriptomics. This technology builds on the capabilities of seqFISH, which can detect and quantify mRNA transcripts at single-cell resolution in intact tissues [41]. Leveraging a combinatorial FISH method—similar to MERFISH—seqFISH+ achieves efficient transcript detection with exceptional spatial resolution [11].

SeqFISH+ employs fluorescent probes designed to specifically target complementary RNA sequences in tissue samples. Through sequential rounds of fluorescent hybridization and imaging, the method generates temporal barcodes that represent thousands of transcript locations. These barcodes are subsequently decoded to create a spatial map of cellular activity, visualized as distinct pseudocolored dots [42].

The workflow begins with primary probe hybridization, where gene-specific primary probes bind to target RNA molecules within cells or tissues. Fluorescently labeled readout probes are then hybridized to the primary probes in approximately 80 sequential rounds. Each round decodes specific RNA barcodes through unique pseudocolor combinations. After each round, the readout probes are stripped using formamide buffer and reapplied for subsequent rounds. Imaging is performed with a standard confocal microscope equipped with multiple fluorescence channels. The sequential imaging rounds collectively reconstruct high-resolution spatial maps of gene expression [43]. SeqFISH+ combines high capture efficiency and sensitivity with the ability to profile a large number of genes. This capability enables the identification of cellular subtypes and the prediction of specific cell–cell interaction patterns, offering profound insights into tissue architecture and function [41].

CosMx Spatial Molecular Imager: the CosMx Spatial Molecular Imager (SMI) is a high-resolution imaging-based method developed by NanoString. It enables in situ measurement of RNA and proteins at subcellular resolution within intact tissue samples by performing multiple cycles of nucleic acid hybridization of fluorescent molecular barcodes [44]. The SMI is compatible with FFPE and FF tissues, capturing the expression of over 1000 RNA targets simultaneously from a single slide [45].

The SMI utilizes ISH probes with unique reporter-binding domains, followed by 16 cycles of sequential hybridization with fluorescent reporters. After each hybridization cycle, high-resolution Z-stacked images are captured. RNA decoding is achieved using a 64-bit barcoding scheme with error correction, allowing highly accurate and sensitive mapping of transcripts to single cells and subcellular compartments [44]. This workflow provides unparalleled resolution and multiplexing capability, leading to the widespread application of SMI in various fields such as cancer research [46,47], neuroscience [48], and immunology [49,50] to uncover the molecular mechanisms underlying disease processes.

In situ sequencing (ISS) approaches can be broadly categorized into targeted and untargeted methods. Targeted ISS, such as STARmap, focuses on preselected genes. Padlock probes, as described in the Xenium, are also integral to ISS methods to target specific RNA molecules with high precision and amplify signals through RCA. This approach achieves high detection efficiency and is capable of analyzing SNVs. Conversely, untargeted methods, including FISSEQ and BaristaSeq, provide genome-wide coverage by sequencing cellular RNAs without prior gene selection. These methods enable the imaging thousands of genes within a single sample, but generally exhibit lower detection efficiency due to challenges such as molecular crowding [51].

STARmap (Spatially resolved Transcript Amplicon Readout mapping): STARmap is a targeted ISS methodology that enhances optical transparency and minimizes background noise in tissues [52] through a combination of SNAIL and hydrogel–tissue chemistry. SNAIL, short for “Specific amplification of Nucleic Acids via Intramolecular Ligation”, is a key feature of STARmap that bypasses the reverse transcription step. This is achieved using a pair of primer and padlock probes designed to hybridize specifically to the same RNA molecule. The padlock probe circularizes only when both probes hybridize to adjacent sites on the RNA, enabling RCA to generate nanometer-sized, single-stranded DNA structures known as DNA nanoballs [53]. This approach bypasses the efficiency challenges of cDNA conversion and reduces noise by the addition of the second hybridization step [17].

During RCA, amine-modified bases are incorporated into the DNA nanoballs, facilitating their embedding within a three-dimensional hydrogel scaffold via hydrogel–tissue chemistry. The resulting tissue–hydrogel complex is cleared of unbound proteins and lipids, significantly enhancing tissue transparency and preserving spatial localization [17]. This porous hydrogel structure, containing the amplified rolling-circle products (RCPs), is well-suited for high-resolution 3D volumetric imaging [52]. STARmap also employs SEDAL (Sequencing with Error-reduction by Dynamic Annealing and Ligation), a sequencing strategy designed to minimize errors [53]. The two-base encoding system implemented in SEDAL ensures high signal fidelity and reduces noise during sequencing cycles.

STARmap PLUS overcame the limitation of detecting 1024 genes in STARmap, offering superior detection efficiency and higher spatial resolution with a 95 × 95 × 350 nm voxel size [54]. Ensuring reliable high-throughput spatial transcriptomic analysis, STARmap is applicable to the study of molecularly defined cell types and activity-regulated gene expression, such as in mouse cortex. The method is effective for analyzing intact tissue samples in 3D while preserving the spatial orientation of the cells for 100–150 μm thick sections and a limited number of targets [53].

BaristaSeq (Barcode in situ targeted Sequencing): BaristaSeq represents an advanced iteration of the gap padlock probe-based method, achieving a five-fold increase in amplification efficiency compared with traditional gap-fill padlock ISS on cultured cells [17]. Designed for sequencing cellular barcodes, BaristaSeq is suitable for multiplexed barcode-assisted neuronal projection mapping. This method is compatible with Illumina sequencing-by-synthesis (SBS), offering a higher signal-to-noise ratio (SNR) compared with SBL. By employing a polymerase enzyme without strand displacement activity, BaristaSeq achieves an increased read length of 15 bases, with potential for further efficiency enhancements via protein engineering. Amplicon stabilization through crosslinking minimizes signal loss during heat cycles, contributing to a sequencing accuracy of at least 97% [55].

The workflow begins with reverse transcription of target mRNA into cDNA, followed by hybridization of padlock probes with two complementary arms. The gap is filled using polymerase, and RCA generates DNA nanoballs to amplify the barcode sequence. Stabilized amplicons are then sequenced using Illumina SBS, ensuring high SNR and accuracy [56]. Potential applications of BaristaSeq include barcode-assisted lineage tracing and mapping long-range neuronal projections, providing a robust and efficient approach for targeted in situ barcode sequencing compatible with next-generation sequencing technologies [55].

FISSEQ (Fluorescent In Situ RNA Sequencing): FISSEQ is an untargeted method that enables the simultaneous localization and sequencing of thousands of RNA species within intact cells and tissues [57], compatible with both FF and FFPE tissues [58]. By utilizing tagged random hexamers as primers, RNAs are reverse transcribed into cDNAs while incorporating a mix of modified bases such as aminoallyl dUTP. This allows for covalent crosslinking of the resultant RCA amplicons to the cellular protein matrix, preserving the spatial context of RNA molecules. Through SBL, FISSEQ generates a read length of 30 bases, allowing unbiased profiling of gene expression, RNA splicing, and post-transcriptional modifications [52,57]. Moreover, the method employs a partition sequencing strategy, which assigns unique address codes to DNA nanoballs, allowing selective sequencing of subsets and improving signal discrimination in densely packed regions [17].

FISSEQ demonstrates versatile applications across diverse fields, including developmental biology, disease research and therapeutic areas. In cancer research, it provides insights into early diagnosis, the impact of gene mutations on metastasis, and responses to targeted therapies, while also identifying potential drug targets [57]. FISSEQ enables the mapping of neuronal connections with molecular annotations, facilitating the creation of detailed spatial maps of brain networks and contributing to the understanding of complex neuronal architectures [59]. Its broad applicability to various sample types—such as cultured cells, tissue sections, and whole-mount embryos—has expanded its utility in studying RNA transcription in intricate biological systems. Notable examples include its application to whole-mount *Drosophila* [60] and mouse embryos, as well as brain tissue sections, highlighting its significant contributions to developmental biology and beyond.

**Figure 1:**
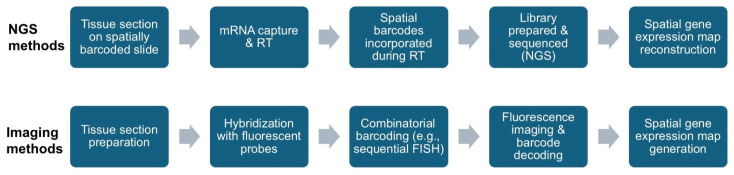
Comparison of spatial transcriptomics workflows.

To highlight the differences in data generation processes between NGS-based and imaging-based spatial transcriptomics platforms, we summarize the typical workflow of each modality in Figure 1.

#### 2.1.3. Recent Advances in Imaging-Based Spatial Transcriptomics

Recent developments in imaging-based spatial transcriptomics have significantly expanded multiplexing capacity and resolution, enabling comprehensive transcriptomic analysis at single-cell and subcellular levels. Two notable advancements are CosMx Whole Transcriptome (WTx) [61] and RAEFISH [24], which demonstrate the highest plex capabilities currently available. CosMx Whole Transcriptome enables the detection of 18,900 protein-coding transcripts using 37,872 imaging barcodes with subcellular resolution. RAEFISH achieves whole-genome coverage by targeting 23,000 human transcripts, while retaining single-molecule spatial resolution in intact tissues. These innovations mark a major step toward achieving a comprehensive view of spatial profiling applicable to a wide range of biomedical research areas.

### 2.2. Spatial Proteomics

While spatial transcriptomics technologies for subcellular DNA and RNA imaging have significantly advanced spatially resolved single-cell biology, an equivalent technology for mapping proteins at the single-molecule level remains elusive [62]. RNAs and their protein counterparts are not expressed in a strict 1:1 ratio due to factors such as RNA degradation, as well as post-transcriptional, translational, and post-translational modifications. Since proteins, in their various forms and modified states, serve as the primary functional units of the cell, directly studying them at the single-cell level is far more informative than relying on transcripts as proxies [63]. These processes can significantly alter the abundance, interactions, and spatial distribution of proteins, highlighting the critical need to directly evaluate proteins, their concentrations, interactions, and precise localization within cells [64]. Achieving a comprehensive understanding of protein organization at the nanoscale level requires addressing four key challenges: sensitivity, throughput, spatial resolution, and multiplexing capacity.

**Figure 2:**
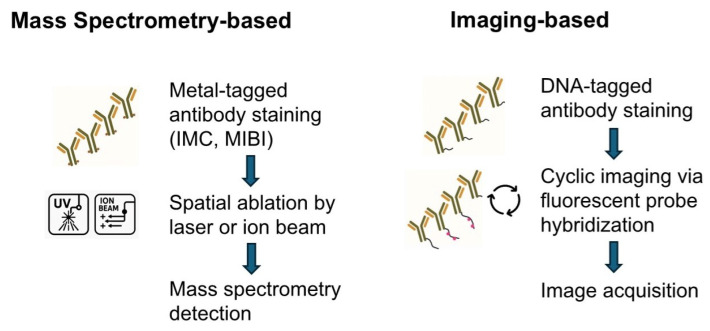
Comparison of spatial proteomics workflows.

**Figure 3:**
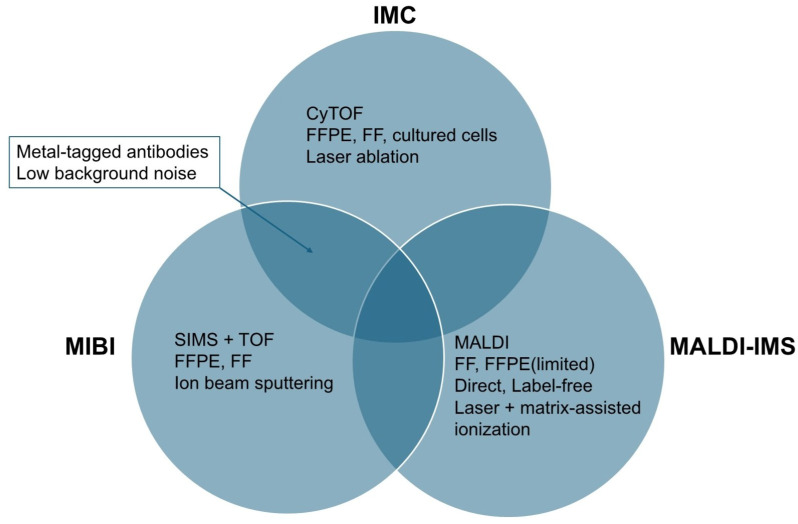
Mass spectrometry-based methods.

Mass spectrometry-based proteomics is currently the gold standard for protein quantification, offering the ability to analyze up to 5000 proteins with single-cell resolution. However, achieving subcellular spatial resolution relies on techniques such as imaging mass spectrometry, multiplexed ion beam imaging, and CODEX, which utilize antibody-based labeling with metal ions or DNA tags. These approaches allow profiling of up to 100 targets within tissue samples at a spatial resolution of approximately 260 nm. Despite these advancements, the majority of proteins measure only 5–10 nm in size, necessitating higher spatial resolution for precise mapping. Recent developments in super-resolution microscopy have greatly improved the ability to investigate protein arrangements at the nanoscale level. However, most of these methods are constrained by significant limitations in their capacity for highly multiplexed imaging [62]. To facilitate a better understanding of the workflow and the comparison between mass spectrometry-based and imaging-based spatial proteomics platforms, we present the general workflow of each platform in Figure 2, and a comparative overview of key mass spectrometry-based techniques is shown in Figure 3.

#### 2.2.1. Mass Spectrometry-Based Proteomics

Mass spectrometry (MS) is a tool for identifying proteins and quantifying their abundance in highly complex mixtures [65]. Mass spectrometry-based methods are highly multiplexed techniques used to capture protein spatial intensity. In this method, mass tags—such as defined molecular masses or metal isotopes—are used as reporter labels on affinity reagents [66].

IMC (imaging mass cytometry): imaging mass cytometry enables the simultaneous detection of up to 40 markers on a single tissue section at the single-cell level in FFPE tissues, frozen tissue sections, and cultured cells [66]. IMC integrates laser ablation with cytometry by time-of-flight (CyTOF) to detect targets labeled with metal-tagged antibodies [67]. Tissue sections undergo a single staining step to minimize technical variability. The laser ablation system then nebulizes tissue pixel by pixel, releasing heavy metal reporter ions, which are subsequently quantified by CyTOF. This allows for single-cell analysis of cell type and state.

A key advantage of this approach is the reduction in noise and channel spillover. Unlike fluorophores, which can exhibit spectral overlap leading to signal “bleed”, metal isotopes have distinct detection peaks. Furthermore, as these heavy metals are not naturally abundant in biological tissues, background noise and autofluorescence are effectively eliminated [64].

IMC employs a region of interest (ROI) selection strategy and supports compartmental segmentation based on biomarker expression. It achieves an initial resolution of 1000 nm, which can be further improved to 260 nm through repeated scans. Despite its strengths, IMC requires meticulous design and validation of complex antibody panels tailored to specific tissues and diseases. Additionally, the tissue ablation process used in IMC renders the sample unusable for further analysis after processing [67].

MIBI (multiplexed ion beam imaging): MIBI achieves subcellular spatial resolution while detecting over 40 markers simultaneously in FFPE or frozen tissues, enabling single-cell segmentation, cell type classification, and spatial analysis of cells, particularly in TMEs [67]. MIBI utilizes mass-tagged antibodies to analyze highly multiplexed protein expression patterns while preserving morphological context. Unlike IMC, which uses a laser, MIBI employs an ion beam to generate secondary ions from the antibodies. These ions are detected using secondary ion mass spectrometry (SIMS), allowing simultaneous imaging of up to 100 targets. The ion beam can precisely image tissues at various depths, enabling z-stack scans and the generation of 3D images with an axial resolution of approximately 250 nm [64]. While MIBI offers significant advantages, non-specific binding between antibodies and epitopes can pose challenges for biomarker validation and standardization. Proper controls are essential for ensuring staining specificity and imaging accuracy [67].

MIBI-TOF is an advanced iteration of MIBI, combining SIMS with time-of-flight (TOF) mass spectrometry for protein quantification, thereby overcoming the limitations of optical imaging [68,69]. This technology uses a primary ion beam to sputter tissues pixel by pixel, releasing secondary ions from the metal-labeled primary antibodies. These ions are then detected and quantified with high precision via TOF mass spectrometry. MIBI-TOF employs isotopically enriched metal reporters conjugated to primary antibodies, enabling the simultaneous, clean quantification of dozens of proteins without the need for secondary antibodies, enzymatic amplification, or cyclical staining. Tissue sections are incubated with all metal-labeled primary antibodies at once, streamlining the protocol and reducing complexity.

During analysis, the primary ion beam liberates the metal tags as secondary ions, which are quantified pixel by pixel using TOF mass spectrometry. This results in high-resolution imaging and precise protein quantification on the same tissue section. The method routinely quantifies over 40 protein targets and is currently being scaled to detect 60 or more. Furthermore, MIBI-TOF is compatible with FFPE samples and can detect both low- and high-abundance proteins across a dynamic range of six orders of magnitude [69]. By combining high-resolution imaging, robust multiplexing capabilities, and a streamlined workflow, MIBI-TOF represents a powerful approach for spatial proteomic analysis in clinical and translational research.

MALDI-IMS (Matrix-Assisted Laser Desorption/Ionization Imaging Mass Spectrometry): MALDI-IMS is one of the most widely used IMS techniques for proteomic applications. This technique enables the direct acquisition of proteomic profiles from thin tissue sections. As a label-free method, MALDI imaging can visualize the distribution of hundreds of molecular compounds in a single measurement, while preserving the morphological and molecular integrity of the tissue [70].

MALDI-IMS combines the sensitivity and selectivity of mass spectrometry with spatial analysis, offering unbiased visualization of the spatial arrangement of biomolecules within tissue [71]. This technology does not require affinity reagents, molecule-specific tags, or chemical modifiers, and directly profiles a large number of biomolecules, including metabolites, lipids, peptides, and proteins, simultaneously [72]. Unlike traditional histological methods, MALDI-IMS is a label-free approach that does not rely on prior knowledge of tissue composition or antibodies, making it a powerful tool for unbiased molecular discovery. By providing spatially resolved molecular information, MALDI-IMS complements and extends conventional histology and immunohistochemistry [73].

A typical MALDI-IMS workflow begins with flash-frozen tissue being cut into thin sections, typically 5–20 µm thick. These sections are then mounted onto a conductive MALDI plate through thaw mounting or mechanical adhesion techniques, avoiding embedding media like sucrose or polymers, which inhibit ion production. Following mounting, the tissue sections are coated with a MALDI matrix, applied using methods such as spray-coating or microspotting. The matrix facilitates the desorption and ionization of molecules in the tissue during laser irradiation. The prepared sample is placed inside the mass spectrometer, and specific regions of the tissue are irradiated by a laser in a predefined rectangular x, y grid. Mass spectra are generated at discrete spatial points, with each spectrum representing an independent molecular profile of the corresponding area of the tissue. These grid points function as “pixels”, each containing thousands of molecular signals of measured mass and intensity. The intensity of a specific signal, or a combination of signals, is plotted on the coordinate system, generating spatially resolved ion distribution maps over the tissue surface [71,74].

MALDI-IMS enables the acquisition of molecular data directly from tissue sections and generates hundreds of molecular distribution maps in a relatively short time. This method allows for the discovery of protein markers that are highly localized to specific tissue structures or transiently expressed during developmental stages. However, sample handling and preparation are critical to preserving the spatial integrity of molecular distributions. Any degradation between sample collection and analysis can compromise results. Ideally, samples are frozen immediately after collection, stored appropriately, and sectioned just before analysis. Mechanical or chemical interferences, such as crosslinking from formalin fixation, can inhibit ionization, although efforts to adapt MALDI-IMS for chemically fixed tissues are showing promise [74].

#### 2.2.2. Imaging-Based Proteomics

CODEX (CO-DEtection by Indexing): CODEX is a multiplexed tissue imaging platform that employs DNA-conjugated antibodies to achieve highly detailed single-cell and spatial analysis of tissues. By utilizing cyclic addition and removal of fluorescently labeled DNA probes hybridized to tissue-bound antibodies, CODEX allows for the simultaneous visualization of up to 60 protein markers on a single tissue slide [75,76]. This technology is compatible with both FFPE and FF tissues, making it versatile for a variety of research and clinical applications [77]. This capability significantly enhances the deep phenotyping potential of flow and mass cytometry, adapting these technologies for use on standard three-color fluorescence microscope platforms to image solid tissues.

CODEX enables highly multiplexed, single-cell quantification of membrane protein expression in densely packed lymphoid tissues, which were previously challenging to analyze. The system combines polymerase-driven incorporation of dye-labeled nucleotides into DNA tags of oligonucleotide-conjugated antibodies with an image-based expression estimation algorithm. This integration ensures precise quantification of marker expression while preserving spatial context, a key feature for studying tissue architecture and cellular interactions. By leveraging multidimensional marker expression and positional data, CODEX enables automatic delineation of cell types, facilitating in-depth characterization of cellular niches and their dynamics during autoimmune disease progression. The rich multivariate datasets generated by this approach provide a valuable resource for advancing image analysis techniques, mapping tissue architecture, and detecting rare cell types [78].

The CODEX workflow starts with antibody conjugation, which involves the attachment of DNA oligonucleotides to purified antibodies, with validation performed using techniques like flow cytometry. Then, tissue preparation includes antigen retrieval, fixation, and antibody staining protocols for FFPE or FF tissues. Cyclic imaging entails the iterative addition, hybridization, and imaging of fluorescent DNA probes, followed by chemical stripping and repetition for all markers in the antibody panel. Finally, data analysis processes the resulting images for single-cell segmentation and marker quantification. Advanced computational methods, such as X-shift clustering and machine learning, are applied to analyze spatial relationships and identify cell types [75].

CODEX has been applied in various areas of research, including cancer, autoimmunity, and infection. In cancer research, CODEX has been used to study the TME in colorectal cancer, identifying two subsets of tumors—those with tertiary lymphoid structures at the invasive front, which correlate with better prognosis, and those with diffuse inflammatory infiltration [79]. In autoimmune diseases, CODEX has been applied to ulcerative colitis to identify inflammatory microenvironment and cellular neighborhoods that persisted despite treatment with TNFα inhibitors. This spatial analysis has helped to model resistance to therapies and has highlighted changes in adaptive immune interactions that correlate with treatment outcomes [80]. In kidney injury, CODEX has been used to map immune–epithelial interactions, revealing distinct anatomical distributions of immune cells, such as the location of neutrophils in the renal medulla and macrophages to the renal cortex, providing insights into spatially coordinated immune responses [81].

This ability to combine high-resolution spatial insights with comprehensive protein expression profiling underscores CODEX’s value as an essential tool for advancing pathology. By enabling detailed investigations into tissue organization and cellular interactions, CODEX offers transformative potential for exploring diverse health and disease contexts, as demonstrated through its applications in cancer research, autoimmune diseases, and tissue injury studies [77].

SUM-PAINT (Secondary-label-based Unlimited Multiplexed DNA-PAINT): SUM-PAINT is a high-throughput nanoscale protein imaging technique with sub 5 nm resolution. By decoupling DNA barcoding from imaging, it enables virtually unlimited multiplexing and reduces imaging time compared with methods like Exchange-PAINT [82], visualizing up to 30 protein targets in neurons within hours. Combining dimensionality reduction techniques, such as UMAP-based clustering [83], facilitates complex dataset analysis.

It has been used to map 30 proteins in neurons, revealing spatial organization and identifying a mixed synapse subtype with excitatory (VGlut1+) and inhibitory (Gephyrin+) components. SUM-PAINT provides nanoscale insights into synaptic proteins like Bassoon and Homer1, advancing understanding of synaptic architecture and plasticity.

Technically, it employs DNA barcoding with signal extinction via strand displacement, ensuring accuracy. While validated on DNA origami and neuronal models, it depends on high-quality antibodies and is currently limited to in vitro samples. Adapting it for intact tissues or complex systems remains a challenge, and large datasets require advanced computational tools. SUM-PAINT has potential for studying protein interactions, synaptic plasticity, and disease processes. Integration with tissue imaging methods like light-sheet microscopy could expand its applications, representing a significant advancement in spatial proteomics and neuroscience [62].

CyCIF (Cyclic Immunofluorescence): CycIF is a straightforward and adaptable method for highly multiplexed imaging, alternating four-color staining with chemical inactivation of fluorophores to progressively build multichannel images. By using standard reagents and equipment, CycIF remains cost-effective and is particularly well-suited for high-throughput assays and screening applications [84].

This open-source technique utilizes a standard epifluorescence microscope and commercially available antibodies to generate images with up to 30 channels through sequential imaging cycles. Each cycle typically involves imaging 4 to 6 channels, followed by fluorophore inactivation.

The most commonly used protocols include staining fixed cells with antibodies directly conjugated to Alexa Fluor dyes. Direct detection is preferred because it allows for greater multiplexing compared with indirect methods, which are constrained by the isotypes and species reactivity of secondary antibodies. Without protease-mediated antibody stripping, indirect immunofluorescence is typically limited to one primary species or isotype per experiment. After staining, fixed cells are imaged in four colors, and the fluorophores are chemically inactivated using a mild base combined with hydrogen peroxide and light. This process preserves cell morphology and improves the signal-to-noise ratio with successive imaging rounds, enhancing data quality.

Unlike traditional antibody-stripping methods, CycIF is a gentle technique, particularly suitable for monolayers of cultured cells. While direct detection is most commonly used, a protocol variant employs indirect immunofluorescence for specific staining needs. Another variant enables the chemical inactivation of genetically encoded fluorescent proteins, allowing researchers to combine multiplexed immunofluorescence with live-cell imaging in cells expressing fluorescent reporter proteins. Together, these protocols highlight CycIF’s versatility, making it a powerful and flexible tool for multiplexed imaging across a range of applications [85].

### 2.3. Spatial Epigenomics

Recent advancements in spatial epigenomics technologies have revolutionized our ability to study the spatial organization of epigenetic regulation within complex tissues, providing transformative insights into gene regulation during development and disease. By applying these techniques to mouse embryos and brains, researchers have uncovered unexpected interrelationships between epigenetic regulation and gene expression across distinct brain regions, shedding light on spatial epigenetic activation, differentiation, and tissue-specific gene regulation [86].

Understanding the spatial organization of epigenetic properties, such as modifications marking active enhancers and promoters, is critical for deciphering how the epigenome shapes cell type development and regulates cell states. During embryonic brain development, morphogenic gradients and transcription factors establish intricate spatial patterns, resulting in neural progenitors that differentiate into diverse neuronal and non-neuronal cell types. Enhancers play a pivotal role in fine-tuning these broad spatial patterns, forming specific domains that give rise to distinct neuronal subtypes. Similarly, in the adult brain, neurons across different cortical layers exhibit distinct chromatin accessibility and epigenetic modification profiles, reflecting their functional and structural diversity.

Despite identifying thousands of epigenetic elements in the brain, their spatial distributions remain unclear. Current methods struggle to resolve short regulatory elements with sufficient precision, underscoring the need for high-resolution spatial profiling to advance our understanding of epigenomic regulation in native contexts [87].

**Figure 4:**
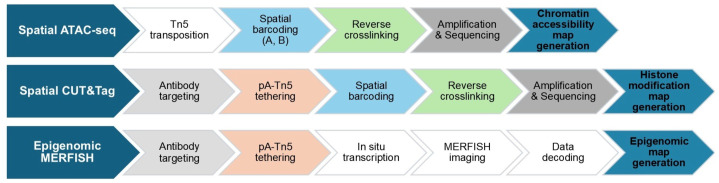
Comparison of workflows for spatial ATAC, CUT&Tag, and Epigenomic MERFISH.

To address these challenges, technologies like spatial-CUT&Tag and Epigenomic MERFISH have been developed to profile histone modifications, while spatial ATAC-seq is specifically designed to spatially profile chromatin accessibility [88]. Furthermore, 3D genome organization can be detected by other imaging-based methods such as chromatin tracing and Hi-M. In the following sections, we will provide an in-depth introduction to these three technologies, outlining their principles, workflows, and contributions to the field of spatial epigenomics. The simplified workflow is illustrated in Figure 4, and Table 3 summarizes a comparison of the three technologies.

Spatial ATAC-seq (spatial Assay for Transposase-Accessible Chromatin using sequencing): spatial ATAC-seq is designed to map chromatin accessibility in tissue sections while retaining spatial resolution at the cellular level. This technique integrates in situ Tn5 transposition with spatial barcoding, enabling genome-wide profiling of open chromatin states, nucleosomes, and TF binding sites in their native tissue context [88].

The workflow begins with in situ Tn5 transposition, where DNA adapters are inserted into accessible chromatin loci within fixed tissue sections. Spatial barcodes are then introduced using a microfluidic barcoding system, which flows two sets of DNA barcodes (A1–50 and B1–50) in perpendicular channels, creating a grid of spatially resolved chromatin fragments. These fragments are subsequently reverse-crosslinked, amplified, and sequenced, allowing the chromatin accessibility data to be mapped back to their original spatial locations. Imaging the tissue section further enables integration of tissue morphology with the spatial map of chromatin accessibility [88,89].

Spatial ATAC-seq achieves high-resolution chromatin accessibility profiling at near-cellular resolution (20 μm) and has been applied to diverse tissues to uncover distinct chromatin accessibility landscapes corresponding to specific cell types and tissue regions. For example, in mouse embryos, it identified region-specific chromatin accessibility patterns related to developmental processes, while in the human brain and tonsils it resolved cell-type-specific chromatin states, providing insights into neural and immune cell organization. This technology is highly reproducible and compatible with other multi-omics data, such as scRNA-seq and histone modification profiling. Despite challenges such as refining spatial resolution and further integrating with other spatial omics approaches, spatial ATAC-seq offers immense potential to transform research in developmental biology, neuroscience, immunology, and clinical pathology by enabling the study of regulatory elements within their native spatial and biological contexts [89].

Spatial-CUT&Tag (Spatial Cleavage Under Targets and Tagmentation): Spatial-CUT&Tag is a groundbreaking technique for genome-wide profiling of chromatin modification states at cellular resolution within intact tissue sections. This method enables spatial profiling of chromatin modifications at the cellular level by combining specific antibody targeting with enzymatic tethering [88]. It integrates in situ CUT&Tag chemistry, microfluidic deterministic barcoding, and next-generation sequencing to create spatially resolved epigenomic maps of histone modifications [90].

The workflow begins with tissue fixation, followed by the application of antibodies targeting specific histone modifications. A secondary antibody is then used to enhance the tethering of protein A coupled to a Tn5 transposase (pA–Tn5) fusion protein to histone marks. Upon activation, the Tn5 transposase inserts adapters containing ligation linkers into genomic DNA at antibody-recognized histone modification sites. Spatial barcoding is achieved by sequentially flowing two sets of barcodes through perpendicular microfluidic channels, creating a two-dimensional grid of spatially barcoded regions (pixels) with resolutions as fine as 20 μm. The inserted adapters efficiently capture spatial information through ligation of the barcodes, allowing the generation of high-resolution fragment libraries. These DNA fragments are reverse-crosslinked, amplified, and sequenced, enabling chromatin modification profiles to be mapped back to their original spatial locations within the tissue [88,90]. Spatial-CUT&Tag targets key histone modifications, including H3K27me3 (repressive chromatin), H3K4me3 (active promoters), and H3K27ac (active enhancers and promoters), offering detailed insights into chromatin organization and regulatory landscapes [88].

Spatial-CUT&Tag has been applied to various tissues, such as mouse embryos and brains, to uncover region-specific chromatin modification patterns linked to tissue-specific functions. In mouse embryos, it revealed chromatin silencing and activation in developing organs like the liver and brain, while in mouse brains it identified chromatin states associated with neuronal subtypes, cortical layers, and radial glial cells. Integration with scRNA-seq further enhanced cell-type identification and developmental trajectories. With near-cellular resolution, it enables chromatin profiling of individual nuclei within complex tissues, providing high-resolution, genome-wide insights into chromatin organization and the spatial epigenetic landscape of tissues. Despite some limitations, such as background noise in frozen tissue sections and limited compatibility with FFPE samples, Spatial-CUT&Tag’s high spatial resolution, genome-wide coverage, and compatibility with spatial omics technologies make it a powerful tool for studying epigenetic regulation in development, homeostasis, and disease. It holds great promise for advancing research in neuroscience, developmental biology, and clinical pathology [90].

Epigenomic MERFISH: Epigenomic MERFISH is an imaging-based spatial omics technique that combines CUT&Tag with MERFISH imaging to enable spatially resolved profiling of epigenetic modifications at single-cell resolution [91]. This approach bridges the gap between traditional epigenomics and spatial transcriptomics by preserving spatial context while interrogating active and inactive promoter and enhancer histone marks in intact tissue sections [92]. By targeting specific histone modifications, such as H3K4me3, H3K27ac, and H3K27me3, Epigenomic MERFISH provides unparalleled insights into chromatin organization, regulatory landscapes, and their roles in gene expression.

The process begins with tissue fixation and permeabilization to preserve spatial architecture, followed by the application of primary antibodies to target histone modifications of interest. Secondary antibodies tether protein A fused with Tn5 transposase to the primary antibodies, enabling the transposase to tag chromatin regions near the targeted histone modifications with DNA adapters containing T7 promoters and sequencing primers. The tissue is then embedded in a polyacrylamide gel for stabilization and digested with proteases to remove proteins. During in situ transcription, T7 RNA polymerase amplifies the tagged DNA regions into RNA, which is hybridized with fluorescently labeled barcoded probes. Finally, sequential rounds of multiplexed MERFISH imaging enable decoding of the spatial distribution of epigenomic loci, generating high-resolution spatial maps with sub-nuclear precision [87,88].

Epigenomic MERFISH has been applied across various tissues, providing insights into spatially distinct chromatin modification patterns. In mouse embryos, it identified region-specific distributions of active promoters and enhancers, uncovering enhancer hubs linked to key developmental genes. In adult mouse brains, it mapped layer-specific chromatin states in the cortex, shedding light on the spatial regulation of neuronal subtypes and cortical organization. Additionally, it demonstrated that active chromatin marked by H3K4me3 and H3K27ac is often located closer to nuclear speckles than repressive chromatin, aligning with previous findings [87].

Epigenomic MERFISH achieves sub-nuclear spatial resolution and genomic resolution below 1 kilobase, surpassing the spatial precision of methods like Spatial-CUT&Tag. Its high multiplexing capacity allows for the simultaneous profiling of hundreds to thousands of epigenomic loci, making it well-suited for large-scale studies. Moreover, its compatibility with transcriptomic MERFISH and other spatial omics technologies enables multi-omics integration, facilitating the direct correlation of epigenetic modifications with transcriptional activity [91].

Despite its strengths, Epigenomic MERFISH has some limitations. As a targeted approach, it requires prior knowledge of loci of interest, restricting its applicability for untargeted genome-wide profiling. While its detection efficiency (35%) is an improvement over earlier methods, further optimization is needed for a more comprehensive view of chromatin regulation [91]. Nevertheless, Epigenomic MERFISH holds immense potential for advancing epigenomic research. Its ability to simultaneously detect multiple epigenetic marks, such as H3K4me1 and DNA methylation, could provide a more comprehensive understanding of chromatin states. Its capability to map epigenetic changes in intact tissues makes it a valuable tool for studying diseases, including cancer, neurological disorders, and developmental anomalies [87].

Chromatin tracing, Hi-M: spatial epigenomics also focuses on the three-dimensional (3D) genome organization within intact cells and tissues. The genome is hierarchically organized into complex structures, and changes in this architecture are closely associated with gene expression. Understanding how the genome is spatially organized provides crucial insights into gene regulation and cellular function [93]. Chromatin tracing and Hi-M are innovative imaging-based techniques that offer direct visualization of chromatin organization at different scales.

Chromatin tracing enables direct 3D visualization of chromatin folding along individual chromosomes in single cells [94]. This technique employs multiplexed sequential FISH to precisely locate genomic loci in 3D space, reconstructing chromatin folding paths with nanoscale accuracy. Chromatin tracing can be applied at the megabase scale to visualize chromosome territories and compartments, or at the kilobase scale to resolve fine topologically associating domains (TADs) and chromatin loops such as promoter–enhancer interactions [95].

Hi-M (High-throughput imaging-based Mapping) combines multiplexed DNA FISH with RNA FISH to visualize 3D chromatin structures and transcriptional activity in a single nuclei. This method has been applied to capture dynamic changes in chromatin organization during developmental processes, such as zygotic genome activation in *Drosophila* embryos [96].

These imaging-based methods complement sequencing-based approaches like Hi-C [97] by providing direct visualization of spatial genome structures without population averaging or computational inference. Their ability to integrate multimodal data makes them invaluable for advancing our understanding of epigenetic regulation in health and disease.

## 3. Spatial Omics Data Analysis Tools

**Figure 5:**
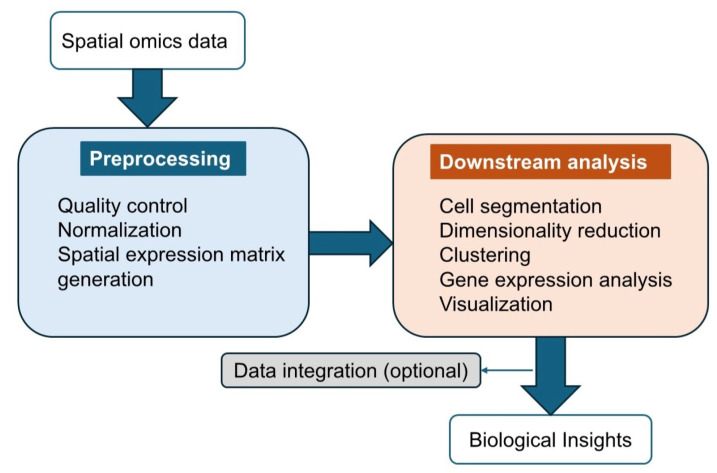
Spatial omics data analysis workflow.

After exploring various technologies for spatial omics data collection, we now shift our focus to the tools used to analyze spatial omics data. Since spatial omics technologies generate large datasets, encompassing tens to thousands of genes and proteins across numerous samples, they are often influenced by technical and biological variability. To derive meaningful insights, robust analytical workflows are essential [98]. Methods for analyzing spatial data typically follow a similar structure, consisting of data preprocessing and downstream analysis. A simplified overview of this pipeline is illustrated in Figure 5.

Data preprocessing includes quality control and normalization to enhance data quality, ensuring more reliable biological insights during downstream analysis [99]. This process can be broadly categorized into imaging-based methods and NGS-based spatial barcoding methods. Imaging-based methods offer subcellular resolution but require complex preprocessing steps such as stitching, segmentation, and signal decoding to create a cell-by-feature matrix. While AI and deep learning have improved segmentation, challenges like low marker specificity persist. In contrast, NGS-based methods rely on genomics-like workflows to map sequencing reads to spatial coordinates and assign biomolecule IDs. These methods are more streamlined but typically provide area-level rather than single-cell resolution, unless combined with high-resolution techniques like Seq-Scope [100] or PIXEL-seq [101]. Both approaches share common downstream steps, which facilitate the extraction of meaningful insights from spatial omics data [4].

Downstream analysis employs various computational and statistical approaches to uncover spatial patterns, cellular interactions, and functional implications [52]. Accurate cell segmentation is a critical step in this process, especially for imaging-based methods, as it delineates cell boundaries using features such as DNA staining for nuclei and membrane or cytoplasmic markers. Recent advancements in segmentation strategies, such as optimized nuclear expansion distances and adaptive tools like Cellpose [102], have shown improved accuracy in assigning transcripts to individual cells. This process defines single-cell or subcellular regions, enabling the assignment of spatial coordinates and molecular profiles to individual cells. Furthermore, tailoring segmentation approaches to specific cell types and tissue structures is important to avoid misassignment of molecular signals [103]. Proper segmentation ensures the quality of downstream analysis by providing reliable input data for subsequent computational steps [104].

Following segmentation, dimensionality reduction methods such as PCA, t-SNE, and UMAP are used to reduce the complexity of high-dimensional data while preserving biologically meaningful variation. Clustering algorithms then identify regions or spots with distinct molecular features, helping to classify cell types or TMEs. Then, gene expression analysis is performed, including differential expression analysis to identify genes with significant differences in expression across clusters or spatial regions, and spatially variable gene analysis to detect genes with spatially dependent expression patterns. For methods such as Visium or Slide-seq, where sequencing units often contain more than one cell, spatial decomposition algorithms infer the proportions of various cell types and assign spatial locations to these cells. Gene imputation techniques further enhance these datasets by predicting low-expressed or missing genes in spatial data [99].

Understanding cell–cell communication is another integral aspect of downstream analysis. This process deciphers how cells interact within tissue microenvironments and how these interactions change under specific conditions, such as cancer or inflammation. Analyzing global cell–cell communication not only deepens our understanding of tissue dynamics but also informs the development of targeted therapeutic strategies [76]. Personalized analyses tailored to specific research objectives are also achievable using a variety of computational tools. Future advances in integrating spatial omics data with patient-specific metadata could accelerate biomarker discovery and personalized therapy development [105].

The integration of spatial omics data with other omics modalities, such as proteomics or metabolomics, enables a more comprehensive understanding of biological systems. Nevertheless, multimodal data integration poses analytic challenges, due to data heterogeneity, batch effects, and missing values. Harmony is a robust and scalable algorithm that integrates multi-datasets by projecting cells into a shared embedding where cells group by cell type, while correcting batch effects and preserving spatial information [106]. MOFA+ further addresses these challenges through variational inference, allowing the integration of large-scale datasets and the modeling of variation across diverse data modalities via flexible sparsity constraints [107].

Integrating single-modality datasets across samples and patients also remains challenging due to technical variability, biological heterogeneity, and differences in spatial resolution. To address these limitations, recent computational tools such as BANKSY [108] and NicheCompass [109] have been developed. BANKSY is an algorithm that integrates spatial and molecular features through neighborhood-aware embeddings, enabling batch-corrected clustering across samples. NicheCompass employs graph-based deep learning to model cell–cell interactions and learn spatially informed cell embeddings, facilitating the alignment and comparison of spatial niches across datasets.

Effective visualization tools, including spatial heatmaps and cell or spot annotation plots, facilitate the interpretation and communication of complex spatial data, making them more accessible to researchers. These visualizations play a pivotal role in hypothesis generation, validation, and communication of findings [99].

A wide range of analysis tools are employed at each stage of the process, addressing different aspects of single-cell and spatial omics data analysis. This section highlights some of the most widely used tools, including Seurat, Scanpy, Giotto, Squidpy, and SpaGCN, and provides a detailed explanation of their applications, ranging from data integration and visualization to advanced spatial analysis leveraging machine learning techniques. Lastly, the role of computational pipelines in spatial omics analysis is discussed.

Seurat is an R package designed for quality control, analysis, and visualization of data, with advanced capabilities for integrating datasets across modalities, such as scRNA-seq and spatial transcriptomics. It achieves integration using “anchors”, which harmonize datasets, correct batch effects, and construct reference atlases. These atlases facilitate the transfer of metadata and cell-type labels to new datasets and enable cross-modality label transfer and spatial mapping, allowing for transcriptome-wide imputation of spatial gene expression patterns [110]. The latest version, Seurat v5, introduces significant updates including a new assay structure using layers, which improves memory efficiency and compatibility with large datasets. It enhances data integration by operating in low-dimensional space. Seurat v5 also allows faster differential expression by integrating with the presto package. SCTransform v2 is now the default normalization method and pseudobulk analysis is streamlined using new aggregation tools. These features, combined with Seurat’s robust tools for multimodal data analysis, visualization, and integration, make it a vital tool for analyzing complex multimodal single-cell data across diverse applications [111].

Scanpy is a Python-based, scalable toolkit designed for comprehensive single-cell analysis, offering functionality for preprocessing, visualization, clustering, trajectory inference, differential expression testing, and gene regulatory network simulations [112]. Specifically optimized to handle large datasets exceeding one million cells by enhancing its analysis speed, 5–90 times faster than Seurat, Scanpy is an ideal choice for large-scale analyses, such as studies like the Human Cell Atlas. The toolkit introduces the anndata object, a generic class for managing annotated expression matrices, which supports modular, memory-efficient pipelines and seamless integration with Python’s scientific ecosystem, including TensorFlow for advanced machine learning applications [113]. Scanpy also supports diverse data formats, such as CSV, HDF5, and loom files, enhancing cross-platform compatibility. Furthermore, it incorporates the PAGA algorithm for computing global topology and visualizing the connectivity structures of complex manifolds, solidifying its position as a powerful and versatile tool for large-scale single-cell omics research [52].

Squidpy is a Python-based framework built on top of Scanpy and anndata, inheriting their modularity and scalability [114]. It is designed for the scalable analysis of spatial transcriptomics and proteomics, leveraging two primary data representations: spatial graphs, which encode relationships among cellular or tissue locations, and large-source tissue images. Squidpy supports a wide range of spatial technologies and provides tools for identifying spatial patterns, quantifying tissue organization, and integrating molecular data with high-resolution images. Its modular infrastructure integrates with Python’s data science ecosystem, enabling interactive visualization, morphology analysis, and efficient computational workflows for large datasets [115].

Giotto is a comprehensive R-based, open-source toolbox for spatial data analysis and visualization, offering a seamless end-to-end workflow. Its analysis module implements a wide range of algorithms to characterize tissue composition, spatial expression patterns, and cellular interactions, while also supporting the integration of scRNA-seq data for spatial cell-type enrichment analysis. The visualization module provides an interactive, user-friendly platform for visualizing analysis results and imaging features [116]. It consists of two main modules: Giotto Analyzer, which provides robust tools for spatial data preprocessing, clustering, dimensionality reduction, and spatial neighborhood analysis, and Giotto Viewer, a web-based application for dynamic visualization in both physical and expression spaces. Giotto is compatible with multiple spatial technologies, including seqFISH+, Visium, and CODEX, and enhances spatial analysis through integration with scRNA-seq data. Key functionalities include spatial gene detection, cell-type enrichment analysis, and mapping of neighborhood interactions. Its scalability, modular design, and compatibility with diverse data types make Giotto a powerful tool for understanding tissue organization and cellular interactions in their native spatial context [117].

Advanced machine learning models, particularly deep learning-based methods, have significantly improved the identification of spatial domains, with SpaGCN being a prime example [7]. SpaGCN is a graph convolutional network (GCN)-based deep learning method designed for analyzing spatially resolved transcriptomics data by integrating gene expression, spatial location, and histological features. By leveraging graph convolution, it constructs a weighted graph to model spatial dependencies among tissue spots, enabling the identification of spatial domains that align coherently in both gene expression and histology. Additionally, SpaGCN identifies spatially variable genes within these domains, offering biologically interpretable insights into spatial gene expression patterns. Compatible with diverse platforms such as Visium, MERFISH, and STARmap, SpaGCN’s AI-driven approach outperforms traditional methods in achieving spatial coherence. Its scalability, versatility, and ability to incorporate histological data make it a powerful tool for advancing spatial transcriptomics research [118]. Future AI-driven approaches could focus on predictive modeling to infer cell–cell communication networks or simulate tissue dynamics under different conditions.

In addition to standalone packages, pipelines have emerged as essential tools for spatial omics data analysis, providing standardized and automated workflows that transform raw data into interpretable results. These pipelines emphasize reproducibility, portability, and standardization, ensuring consistency across different datasets and experimental conditions. One of the most widely adopted frameworks is nf-core, a community-curated ecosystem of pipelines built using the Nextflow workflow language. nf-core pipelines are version-controlled, containerized, and subjected to continuous integration testing, ensuring reproducible and scalable across diverse computing environments [119]. A notable example is nf-core/spatialvi, which streamlines the analysis of Visium spatial transcriptomics data. This pipeline automates raw data processing using Space Ranger and preprocessing steps, then incorporates the spatialvi model [120] to facilitate probabilistic modeling and deconvolution. By encapsulating these steps, it simplifies complex spatial workflows for broad accessibility and reproducibility [121]. Looking ahead, pipelines like nf-core/spatialvi could be expanded to address challenges in multimodal integration and cross-sample harmonization. Such developments would further enhance the utility of spatial omics pipelines in systems biology and translational research.

Spatial omics workflows increasingly adopt the FAIR principles—Findable, Accessible, Interoperable, and Reusable [122]—to ensure reproducibility and collaborative research. The SpatialData framework [123] exemplifies this approach by providing a FAIR storage format and a library designed for Python 3.9 and above, enabling standardized data representation, efficient access and manipulation, and integration with tools such as spatialvi, spatialxi and PRISM [124]. By supporting shared annotation formats and metadata tagging, SpatialData facilitates interoperability and reproducibility across diverse spatial omics platforms. Specifically, spatialvi utilizes SpatialData to store raw count cell-type deconvolution results, and spatial coordinates in a structured hierarchy, while enabling cross-technology integration including Visium, Xenium, and MERFISH. Similarly, PRISM leverages SpatialData to manage multiplexed spatial proteomics imaging data, supporting interactive analysis, cell segmentation, and annotation through a user-friendly graphical interface. The adoption of such FAIR-compliant frameworks addresses key bottlenecks in spatial omics, streamlining analysis, enhancing data sharing, and supporting robust multimodal studies.

## 4. Application in Clinical Research

In this section, we describe the clinical applications of spatial omics technologies across various disease contexts, including cancer, neurological disorders, and autoimmune diseases by highlighting representative studies.

### 4.1. Cancer Research

Solid tumors are composed of subpopulations of cells with distinct genomic alterations within the same tumor, known as intratumoral heterogeneity [125]. This heterogeneity can manifest in two forms: spatial heterogeneity, which refers to the uneven distribution of genetically diverse tumor subpopulations across or within a single tumor or disease site, and temporal heterogeneity, describing dynamic genetic variations within a tumor over time. These variations can result in a non-uniform distribution of distinct tumor-cell subpopulations, influencing disease progression and therapeutic outcomes [126]. This intratumoral heterogeneity has been studied using spatial transcriptomics, providing critical insights into the spatial organization of molecular and cellular features within tumors.

For example, in colorectal cancer, spatial transcriptomics revealed the coexistence of multiple Consensus Molecular Subtypes (CMS1, CMS2, CMS4) within a single tumor. These subtypes were spatially localized, with CMS1 regions enriched with immune cell infiltration, while CMS2 and CMS4 regions exhibited stromal activation and WNT pathway signaling. This spatial distribution highlights functional heterogeneity, emphasizing the diverse roles of tumor regions in progression and therapy response [127].

Similarly, in triple-negative breast cancer, spatial transcriptomics uncovered substantial spatial heterogeneity across 92 patient samples by identifying nine distinct spatial archetypes (SAs). These SAs represent unique spatial arrangements of tumor cells, stromal components, and immune populations, varying significantly across TNBC molecular subtypes such as basal-like, immunomodulatory, and mesenchymal subtypes. This spatial variability underscores the complexity of TNBC tumor biology and its implications for precision medicine [128].

The tumor microenvironment is a complex and dynamic entity composed of cancer cells, immune cells, stromal cells, blood vessels, and an extracellular matrix. It varies across tumor types but plays a critical role in supporting tumor growth, local invasion, and metastasis. The TME fosters reciprocal interactions between cancer cells and its components, promoting survival and adaptation, such as inducing angiogenesis to counter hypoxia and acidity. Diverse immune cells within the TME can exhibit both pro- and anti-tumorigenic functions, making the TME a key focus for identifying therapeutic targets [129]. A detailed understanding of the TME, which is critical for cancer research, requires knowledge of its spatial information, including cell location and cell–cell interactions [130]. Novel spatial omics technologies have emerged as powerful tools to gain in-depth insights into the TME, and several studies demonstrate the utility of spatial omics techniques in uncovering key aspects.

The molecular characteristics and TME of seminomas were investigated using scRNA-seq, scATAC-seq, and spatial transcriptomics. This study identified key transcription factors, such as TFAP2C, POU5F1, SOX17, and NANOG, which play critical roles in tumorigenesis, and demonstrated that TFAP2C promotes tumor migration and invasion. The analysis also uncovered 15 immune cell subtypes within the seminoma TME, revealing an immunosuppressive state driven by T cell exhaustion and macrophage activity, facilitated through extracellular matrix degradation mediated by enzymes like MMP9 and CTSK. Spatial transcriptomics highlights tumor–immune interactions mediated by pathways such as the MIF-CD74/CXCR4 axis, which could serve as therapeutic targets [131].

Another study utilized spatial transcriptomics to analyze the TME of high-grade serous ovarian carcinoma. By comparing tumor samples from patients with a poor response (PR) and an excellent response (ER) to neoadjuvant chemotherapy, the study reveals distinct spatial and molecular characteristics between these groups. PR tumors are dominated by stromal clusters, including myofibroblasts and mesenchymal cells, while ER tumors are enriched with immune cell clusters, such as T cells, B cells, and NK cells, which are associated with better chemotherapy outcomes. The spatial organization of ER tumors is more compact and organized, enhancing immune-mediated responses, whereas PR tumors show dispersed clusters with significant stromal–mesenchymal interactions, contributing to therapy resistance. Spatial transcriptomics further identifies ligand–receptor interactions, such as those distinct to PR and ER groups, underscoring the variability in cell–cell communication within the TME [132].

In prostate cancer research, scRNA-seq and Slide-seqV2 have been employed to dissect the TME and its association with disease progression. The prostate TME exhibits an immune-suppressive landscape characterized by tumor-inflammatory monocytes, M2-like macrophages, exhausted T cells, and increased regulatory T-cell activity, which facilitate immune escape. Stromal populations, including endothelial cells, fibroblasts, and pericytes, show increased angiogenic activity and extracellular matrix remodeling in tumor regions compared with healthy tissues, contributing to tumor vascularization and progression. A distinct “Prostate Tumor Gene Signature” was identified, distinguishing malignant luminal epithelial cells from non-malignant cells. RNA velocity analysis revealed differentiation trajectories for epithelial subpopulations, suggesting progenitor roles in tumor development. Ligand–receptor interaction analysis uncovered tumor–stromal communication pathways, such as VEGF signaling promoting angiogenesis and CCL20-CCR6 signaling driving immune suppression [133].

As described before, immune cells are critical players in the TME, shaping the fate of cancer cells by either promoting or inhibiting carcinogenesis, progression, metastasis, and recurrence. Recent immunotherapy strategies, such as immune checkpoint blockade, adoptive cell transfer, cytokines, and cancer vaccines, have demonstrated promising clinical efficacy by enhancing the antitumor state of the immune system [134]. Spatial omics technologies have emerged as powerful tools for advancing immunotherapy design by providing high-resolution insights into spatial organization and interactions within the TME. For instance, CODEX was employed to explore the TME of patients with non-small cell lung cancer undergoing immune checkpoint inhibitor therapy. The findings revealed how spatial cell–cell interactions, such as proximity between regulatory T cells and CD8+ T cells in non-responders, contribute to immunosuppressive environments. In contrast, responders exhibited macrophage interactions with HLA-DR+ tumor cells, indicative of an immune-active TME. Moreover, spatial clustering identified immune-rich and tumor-dominant neighborhoods, with the presence of effector CD4+ T cells in tumor regions correlating with improved survival outcomes. These insights underscore how spatial omics can unravel immune mechanisms and heterogeneity in the TME, guiding the development of tailored immunotherapies [135].

Tertiary lymphoid structures (TLSs), ectopic lymphoid formations that develop in tissues experiencing chronic inflammation and persistent antigen exposure, are observed in conditions such as autoimmune diseases, chronic infections, graft rejection, and cancers [136]. TLSs have significant potential as prognostic tools in cancer, as their high densities are generally associated with improved patient survival in cancers such as breast, colorectal, and lung cancer. TLSs promote immune responses through the production of effector cytokines by T cells, tumor-specific antibodies by B cells, and enhanced antigen presentation by dendritic cells [137]. In a study on recurrent/metastatic head and neck squamous cell carcinoma, GeoMx DSP was used to investigate the role of TLSs in the efficacy of immune checkpoint inhibitors. The study found that responders exhibited TLSs located closer to tumor cells, with this spatial proximity correlating with improved outcomes. Furthermore, TLSs in responders showed upregulation of interferon signaling and immune modulation pathways, emphasizing their role in enhancing antitumor immunity. These findings highlight the critical importance of TLS spatial organization in shaping TMEs, influencing immunotherapy success, and providing a foundation for improved patient stratification and targeted therapeutic strategies [138].

### 4.2. Neurological Diseases

The brain is one of the most complex living tissues, composed of an exceptional diversity of cell types with unique functional connectivity. Understanding how these various cell types contribute to specific brain functions requires correlating the molecular identities of individual cells, as revealed by scRNA-seq, with their spatial context in intact tissue. Spatial transcriptomics provides powerful insights to resolve the intricate spatial organization of cell types in the brain and their connectivity [139]. By generating detailed gene expression atlases for different brain regions, this approach has advanced our understanding of brain structure and function, enabling the study of brain development, physiology, and disease mechanisms [140].

For instance, a high-resolution atlas of the adult mouse brain was developed by integrating scRNA-seq and MERFISH. This atlas revealed a strong correspondence between cell-type transcriptomic identity and spatial organization. Notably, it uncovered a dichotomy between the dorsal and ventral brain regions: the dorsal brain contains fewer, highly divergent neuronal types, while the ventral brain has more numerous, closely related neuronal types. The study also highlighted extensive diversity in neurotransmitter and neuropeptide expression and identified transcription factors as key determinants of cell-type identity, defined by a combinatorial code across the brain [37]. These insights provide a foundation for exploring the brain’s molecular and cellular organization and its role in various neurological diseases.

One such application is in Alzheimer’s disease (AD) research, where spatial transcriptomics has enabled detailed investigations of transcriptional changes in tissue domains surrounding amyloid plaques. In an AD mouse model, early transcriptional alterations in a 100 µm diameter around plaques revealed enrichment in gene co-expression networks related to myelin and oligodendrocyte genes, suggesting early involvement of oligodendrocytes in AD pathology. In later stages, a multicellular co-expression network of plaque-induced genes emerges, involving pathways related to the complement system, oxidative stress, and inflammation [141]. Further, the *Trem2*^R47H^ mutation, a genetic risk factor for AD, was studied using MERFISH in wild-type and 5xFAD mouse models. This mutation significantly impacts microglia, astrocytes, and neuronal cells, with spatial analysis showing microglia clustering predominantly near plaques. The *Trem2*^R47H^ mutation drives distinct transcriptional profiles in glial and neuronal cells, highlighting its role in neuroinflammation and plaque-associated pathology [142]. In human AD studies, spatial transcriptomics revealed regional gene expression patterns associated with selective vulnerability to amyloid-beta (Aβ) and tau pathologies. Key genes such as *APOE* and *MAPT* are linked to tau vulnerability, while *CNTNAP2* and *TMEM106B* are associated with Aβ deposition. These gene-to-pathology associations correlate with cognitive decline, offering mechanistic insights and potential applications for biomarkers and therapeutic targets. Together, these findings underscore the complementary yet distinct roles of Aβ and tau in AD progression [143].

Spatial omics has also provided valuable mechanistic insight into neurodevelopmental and neuropsychiatric disorders [139], including autism spectrum disorder (ASD) and schizophrenia.

ASD is a neurodevelopmental disorder characterized by deficits in social communication and restricted, repetitive behaviors. Its presentation spans a spectrum of symptoms, ranging from mild to severe, reflecting the heterogeneity of its underlying molecular and cellular mechanisms [144]. One study utilizing Visium on human dorsolateral prefrontal cortex (DLPFC) tissue revealed key spatial and molecular characteristics of cortical cell populations. Genes harboring mutations associated with ASD showed preferential expression in layers 2 (L2) and 5 (L5) of DLPFC. These layer-specific gene expression patterns were linked to clinical characteristics, with genes associated with neurodevelopmental delay predominantly expressed in L2. This demonstrates the power of spatial transcriptomics in uncovering layer-specific molecular patterns, providing deeper insights into the cortical architecture underlying ASD [15]. Another study, which integrated scRNA-seq, ATAC sequencing, and Visium, linked mutations in coding and non-coding regions to cerebellar dysfunction and ASD. Specific ASD-associated genes, such as *CNTNAP2*, exhibited spatiotemporal expression patterns unique to human cerebellar development. *CNTNAP2*, highly expressed in Purkinje cells, was implicated in axon guidance and synaptic signaling, which are essential for cerebellar function. Additionally, the transcription factor HEY1 was found to regulate granule cell precursor proliferation and migration, processes that are frequently disrupted in neurodevelopmental disorders such as ASD [145].

Schizophrenia is a complex mental disorder characterized by a wide range of symptoms, including delusions, hallucinations, disorganized thinking and behavior, flat affect, and amotivation, among others [146]. The cellular and molecular basis of schizophrenia was studied by analyzing the DLPFC using single-nucleus RNA sequencing (snRNA-seq) and Visium. The analysis reveals significant compositional and transcriptomic changes in neurons in the upper cortical layers (layers 2/3), including increased principal neurons and decreased GABAergic interneurons, such as PVALB and SST subtypes. The upper cortical layers also exhibited hotspots of dysregulated gene expression, particularly in pathways associated with synaptic plasticity, energy metabolism, and neurotransmission. These findings highlight the critical role of upper cortical layers in the pathophysiology of schizophrenia, providing new insights into its molecular and cellular underpinnings [147].

### 4.3. Autoimmune Diseases

Autoimmune diseases are chronic conditions characterized by the loss of immunological tolerance to self-antigens [148] and aberrant B cell and T cell reactivity against normal host tissues. These diseases are highly heterogenous, affecting individuals of all ages with a higher prevalence in women. A defining feature of autoimmune diseases is the production of autoantibodies, which serve as biomarkers for diagnosis, classification, and monitoring disease activity [149]. Spatial omics technologies have significantly advanced our understanding of the molecular and cellular mechanisms underlying autoimmune diseases.

In lupus nephritis (LN), spatial proteomics has revealed region-specific immune cell infiltration within kidney compartments. Activated NK cells and neutrophils are predominant in glomeruli, while resting NK cells are prevalent in tubules. Differentially expressed proteins (DEPs) highlight enriched immune pathways in glomeruli, such as complement activation, and metabolic processes in tubules [150]. Furthermore, scRNA sequencing combined with spatial transcriptomics identified APOE+ monocytes/macrophages, a distinct immune subset elevated in LN. These cells, localized around glomeruli and renal interstitium, exhibit reduced antigen-presenting capacity and interferon secretion, suggesting a compensatory anti-inflammatory mechanism. Spatial transcriptomics also uncovered lymphangiogenesis, facilitating the trafficking of APOE+ monocytes to inflamed regions, with APOE identified as a key regulator of immune communication and inflammation [151].

In rheumatoid arthritis (RA) and spondyloarthritis (SpA), spatial transcriptomics has provided insights into synovial tissue-specific mechanisms. RA tissue exhibits overexpression of T cell activation markers (CXCL9, CXCL13) and ectopic lymphoid structures, reflecting its autoimmune nature. In contrast, SpA shows enrichment of mesenchymal and tissue repair pathways, along with markers of extracellular matrix remodeling. Immune cell distributions also differ, with central memory T cells predominant in RA and effector memory T cells prominent in SpA. These findings underscore the role of spatial transcriptomics in dissecting disease-specific immune landscapes [152].

In autoimmune thyroid diseases (AITDs), such as Graves’ disease (GD) and Hashimoto’s thyroiditis (HT), spatial transcriptomics identified poorly differentiated thyroid follicular cells with high CD74/MIF expression, suggesting dysregulated antigen presentation. Different subpopulations of fibroblasts were observed, with ADIRF+ myofibroblasts enriched in GD and inflammatory fibroblasts in HT. GD was associated with angiogenesis and fenestrated vessels expressing PLVAP, while HT showed increased immune infiltration and oxidative stress pathways. These findings highlight the heterogeneity of AITD and suggest therapeutic targets, including the CD74/MIF axis and fibroblast-mediated processes [153].

**Figure 6:**
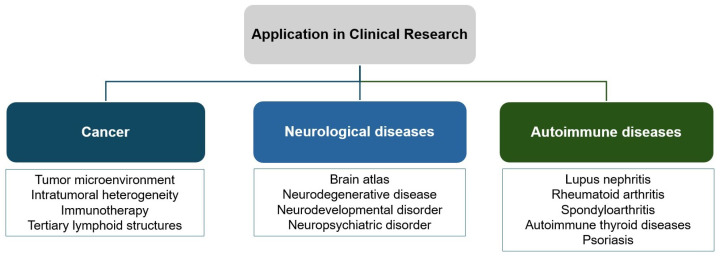
Application of spatial omics in clinical research.

In psoriasis, spatial transcriptomics uncovered three inflammatory niches: the hyperplastic epidermis, papillary dermis, and reticular dermis. The epidermis is enriched in IL-1 cytokines and chemokines (CXCL16, CCL27), driving keratinocyte activation. The papillary dermis contains M2 macrophages, CD4+ T cells, and IL-32/IFN response signatures, while the reticular dermis hosts activated fibroblasts expressing CXCL12, CCL19, and LRRC15. Interestingly, key psoriasis-associated cytokines like IL-23 and IL-17 were sparsely detected, while innate cytokines such as IL-32 and IL-36 dominated, suggesting their central role in psoriatic inflammation [154]. Figure 6 provides a visual summary of how spatial omics technologies are being applied in clinical research in diverse disease domains.

## 5. Guidelines to Design Spatial Omics in Clinical Research

First, study design and sample preparation must clearly define whether the research centers on transcriptomics, proteomics, or epigenomics, ensuring appropriate sample selection between fresh frozen or FFPE tissues according to technology compatibility, while considering sample diversity to capture heterogeneity and optimizing fixation, sectioning, and embedding procedures to preserve molecular integrity, maintain spatial context, and minimize degradation or diffusion effects [155].

Second, a range of specialized techniques is required, including molecular dynamics tools such as imaging mass cytometry (IMC) and multiplexed ion beam imaging (MIBI) for high-plex protein analysis; fluorescence-based methods such as CODEX, SUM-PAINT, and CycIF for antibody-based multiplexed imaging; and spatial epigenetic technologies such as spatial ATAC-seq for chromatin accessibility, Spatial-CUT&Tag for histone modification profiling, and Epigenomic MERFISH for high-resolution epigenetic mapping [156,157,158,159,160].

Third, data acquisition and processing play a crucial role in ensuring the accuracy and reliability of spatial omics analyses by implementing rigorous quality control measures, such as assessing tissue integrity, RNA/protein yield, and contamination, alongside imaging calibration and background noise reduction. Preprocessing steps, including AI-assisted cell segmentation for single-cell resolution and normalization techniques to reduce batch effects, further enhance data consistency. Finally, multi-omics data integration, combining spatial transcriptomics, proteomics, and epigenomics, facilitates a comprehensive understanding of molecular interactions within the tissue microenvironment [123,134,161].

Fourth, computational analysis and interpretation are essential for extracting meaningful biological insights from spatial omics data by employing deep learning-based segmentation tools like Squidpy and Giotto for cell segmentation and annotation, applying dimensionality reduction techniques such as UMAP, t-SNE, or PCA for high-dimensional data visualization, identifying spatially variable genes using machine learning models like SpaGCN, inferring cell–cell communication networks within tissue microenvironments, and enriching biological insights through pathway and functional analysis using Seurat, Scanpy, and CellChat [162,163].

Fifth, validation and reproducibility are critical for ensuring the reliability of spatial omics findings by correlating results with orthogonal techniques such as bulk RNA-seq and IHC, standardizing sample collection, data acquisition, and analysis to enhance technical reproducibility, and performing cross-validation with other spatial omics datasets to confirm consistency [164,165].

**Figure 7:**
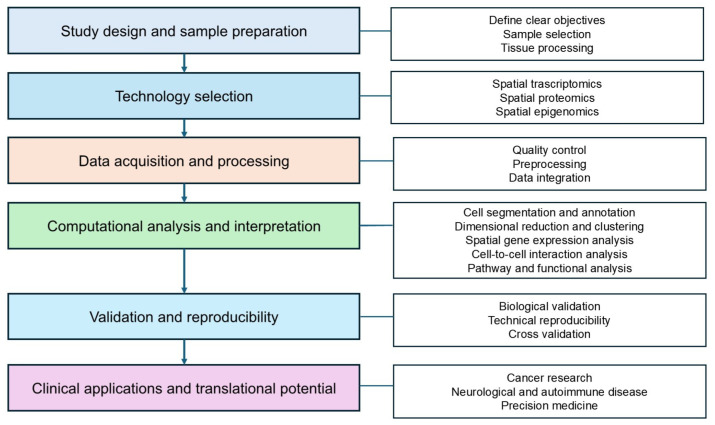
Guidelines to design spatial omics in clinical research.

Finally, clinical applications and translational potential of spatial omics extend to cancer research, where it enables tumor heterogeneity mapping and immune microenvironment profiling, neurological and autoimmune diseases, where single-cell resolution methods provide insights into disease pathology, and precision medicine, where spatial omics is integrated with patient-specific therapeutic strategies to advance personalized treatment approaches. The simplified workflow for the above guidelines is illustrated in Figure 7.

## 6. Conclusions

Spatial omics technologies have emerged as transformative tools in modern biology and clinical research, playing a pivotal role in the decoding of spatial patterns of genes, proteins, metabolites, and epigenomes within tissue microenvironments. The various methodologies discussed in this article provide valuable insights into understanding disease pathophysiology and identifying therapeutic targets, with demonstrated applications in cancer, neuroscience, and immunology.

Specifically, spatial transcriptomics serves as a powerful approach for exploring interactions within complex tissues, such as the TME, while spatial proteomics enables the study of cellular interactions at the protein level. Furthermore, spatial epigenomics and spatial metabolomics technologies contribute to high-resolution analysis of chromatin accessibility and metabolomic profiles, respectively, in native tissue contexts.

Future research is expected to integrate these spatial omics techniques into a multi-omics framework, allowing for deeper biological insights and expanded clinical applications. Overcoming current challenges, such as analytical efficiency and data integration, will further position spatial omics as a cornerstone of precision medicine and personalized therapy. By addressing these limitations, spatial omics technologies hold the potential to revolutionize our understanding of complex biological systems and improve patient outcomes.

## Figures and Tables

**Table 1:** Comparison of NGS-based spatial transcriptomics methods.

Method	Resolution	Tissue Compatibility	Features
**Visium**	55 μm	FFPE, FF	Barcoded spots
**Slide-seq**	10 μm	FF	Barcoded beads
**Stereo-seq**	220 nm	FF	DNA nanoball patterned array chips
**DBiT-seq**	10 μm	FFPE, FF	Microfluidic spatial barcoding

**Table 2:** Comparison of imaging-based spatial transcriptomics methods.

Method	Detection Principle	Spatial Resolution	Genomic Resolution	Tissue Compatibility	Key Applications
Xenium	FISH with RCA	Subcellular	Gene-level (1000+)	FFPE, FF	Cancer research, Brain mapping, TME analysis
MERFISH	Combinatorial barcoding + sequential FISH	Single-molecule	Gene-level (1000+)	FF, Cultured cells	Neuroscience, Developmental biology, Cancer research
SeqFISH+	Sequential FISH with temporal barcoding	Subcellular	Gene-level (10,000+)	FF, Cultured cells	Cell-type identification, Tissue architecture, Cell–cell interactions
CosMx SMI	Combinatorial barcoding + sequential FISH	Subcellular	Gene-level (1000+)	FFPE, FF	Cancer research, Neuroscience, Immunology
STARmap	Padlock probe-based RCA	Subcellular	Targeted genes	FF, Hydrogel-embedded tissues	Brain studies, Molecularly defined cell typing, Gene expression in intact tissue
BaristaSeq	Gap padlock probe-based sequencing	Subcellular	Targeted barcode sequencing	FF, Cultured cells	Lineage tracing, Neuronal projection mapping
FISSEQ	Reverse transcription + RCA + SBL	Subcellular	Genome-wide (untargeted)	FFPE, FF, Cultured cells	Cancer research, Neuronal mapping, RNA splicing analysis

The color code highlights ISH (blue rows) and ISS (orange rows) approaches.

**Table 3:** Comparison of spatial epigenomics technologies.

Method	Focus	Target	Barcoding Method	Resolution
Spatial ATAC-seq	Chromatin accessibility	Open chromatin, TF binding sites	Microfluidic dual-barcode grid	20 μm
Spatial-CUT&Tag	Histone modifications	H3K27me3, H3K4me3, H3K27ac	Microfluidic dual-barcode grid	20 μm
Epigenomic MERFISH	Epigenetic modifications	H3K27me3, H3K4me3, H3K27ac, Enhancer regions	Sequential fluorescent barcoding	Sub-nuclear (1 kb)
Chromatin tracing	3D genome organization	Locus-specific chromatin structure	Multiplexed FISH	Sub-nuclear (30 kb)
Hi-F	3D genome organization	Chromatin contacts (TADs, loops)	Multiplexed FISH	Sub-nuclear (2 kb)

## Data Availability

The data that are discussed in this article are presented in cited studies.
